# A comparison of success rates of introduced passeriform birds in New Zealand, Australia and the United States

**DOI:** 10.7717/peerj.509

**Published:** 2014-08-12

**Authors:** Michael P. Moulton, Wendell P. Cropper

**Affiliations:** 1 Department of Wildlife Ecology and Conservation, University of Florida, Gainesville, FL, United States; 2 School of Forest Resources and Conservation, University of Florida, Gainesville, FL, United States

**Keywords:** Introduced species, Propagule pressure, History, Site-level factors, Introduced species, Establishment, Individual-level factors in introduction success, Passeriform birds, Location-level, Species-level

## Abstract

In this study, we compiled lists of successful and unsuccessful passeriform introductions to nine sites in New Zealand, Australia and the United States. We limited our analysis to introductions during the 19th century to minimize potential variation in transport modes and habitat quality changes, such as those due to increasing urbanization. We compared introduction success rates at three levels. First we included all passeriforms introduced to any of the sites in the three locations, then we compared the fates of just those species with a European origin and finally we compared success rates of just the 13 species released into all three locations. We found that the pattern of success or failure differed significantly across the three locations: Passeriforms introduced by acclimatization organizations to the United States were significantly more likely to fail than those introduced to New Zealand or Australia. Several species that succeeded in either New Zealand or Australia failed in the United States, even after the introduction of seemingly sufficient numbers.

## Introduction

What are the factors that determine whether a species introduction will succeed or fail? Duncan, Blackburn & Sol (2003) suggested that characteristics of the introduced species, the location of the introductions, or features of the introduction events themselves could influence the outcomes of introductions. With this in mind, numerous studies have examined patterns of success in avian introductions in the hope of unraveling the relative roles of these variables (e.g., Cassey et al., 2004; Lockwood, Cassey & Blackburn, 2005; Sol et al., 2005; Sol et al., 2012). A general conclusion of many of these studies was that introduction effort, meaning propagule pressure (an event-level factor), has been the most important consideration (e.g., Blackburn, Lockwood & Cassey, 2009; Sol et al., 2005), and that life-history variables or other species-level factors are secondary in importance. However, in a population model analysis (Cassey, Prowse & Blackburn, 2014) found that a life-history variable (net reproductive rate) of the species was more important than propagule pressure in deciding the outcomes of avian introductions. The notion that differences in success rates could be due primarily to variation among locations where species are introduced has largely been ignored (e.g., Blackburn, Lockwood & Cassey, 2009) despite results from studies such as Case (1996).

Three problems can arise in assessing the importance of location-level effects. First, the available data are highly restricted geographically, with Australia, New Zealand and the United States having had particularly large numbers of introductions (Sol et al., 2012). Second, introduction success rates could differ among locations because of differences in the composition of the species being compared (Sol, 2000). Thus, such comparisons should be limited to sets of species that are at once taxonomically related (i.e., within orders) and share biogeographic origin. Without such restrictions, success rates in different locations could occur independently of any true location-level differences. Finally, historical effects specific to each introduction could influence the outcome of location-level comparisons.

In this context, historical effects could impact introduction success rates in three ways. First, environments, resource levels, and habitats all can, and commonly do, change over time. For example Brooklyn, New York was mostly rural farmland in the late 1800s, when Eurasian Skylarks (*Alauda arvensis*) were introduced there (Phillips, 1928; Long, 1981; Lever, 1987; Lever, 2005). This region of Long Island has since been completely urbanized, and during this profound landscape-level change the Eurasian Skylarks have perished (Eaton, 1914; Phillips, 1928).

The second influence involves the transport phase. Birds introduced in the nineteenth century were carried to the new land by ship, and in the case of European birds brought to distant locations such as New Zealand, this meant a journey of several weeks, which in turn likely affected the health and condition of the birds. Lamb (1964) for instance describes a voyage by the ship *Matoaka* that sailed from England for New Zealand (Lyttleton) and arrived after a 94-day journey. Of the 444 birds that were shipped on that particular voyage, only 166 survived the ordeal (a death rate of more than 60%). Thomson (1922) lists numerous examples of the staggering death rate for birds shipped from England to New Zealand, and Jenkins (1977) lists similar examples for shipments to Australia. For instance among 150 larks shipped to Australia in July 1860, only seven survived, a death rate in excess of 95% (Jenkins, 1977). Thus, it could be misleading to compare any potential influencing factor without first accounting for such historical differences in modes of transport.

A third point involves the length of time since a species was introduced to a new land. Simberloff & Gibbons (2004) noted that some populations of introduced species mysteriously suffered population crashes long after they seemed to have been established. This pattern could be attributed to many factors including, low genetic diversity in the founding population, landscape-level changes in habitat quality, or rare stochastic events (e.g., hurricanes or ice storms).

To control for possible taxonomic variation we limited our analyses to the passeriform birds introduced to nine sites across three locations: Australia (2 sites); New Zealand (4 sites); and the United States (3 sites). Although these scales might seem crude they are the scales used in most studies of avian introduction success patterns (Veltman, Nee & Crawley, 1996; Duncan, 1997; Green, 1997; Cassey et al., 2004; Lockwood, Cassey & Blackburn, 2005; Sol et al., 2005; Sol et al., 2012).

In an effort to reduce any influence of historical factors we limited our analyses to introductions in the last half of the nineteenth century. To deal with the problem of heterogeneity in the species involved in the comparison (Sol, 2000) we conducted our analyses at three levels: all introduced passeriforms in each regions (Australia, New Zealand and the United States); species with a European origin only; and lastly, the subset of just those species introduced to all three regions.

## Materials and Methods

The nine sites we included were: Australia (2)–Victoria, and South Australia (meaning the Adelaide area–Jenkins, 1977); New Zealand (4)–meaning the acclimatization districts of Auckland, Wellington, Canterbury, and Otago; United States (3)–Cincinnati, Ohio; Portland, Oregon; and New York City. For our analyses, we included Manhattan (Central Park) and Brooklyn (Greenwood Cemetery) in the New York City area. These last two sites are separated by less than 25 km. Each of the sites in Australia, New Zealand and the United States had an acclimatization society or comparable institution that was involved in bird introductions.

For each site we tallied the number of successfully introduced species and the rates of success. For the four acclimatization districts in New Zealand and the two in Australia our lists were based on previous compilations (New Zealand–Moulton, Cropper & Avery, 2011; Australia–Moulton et al., 2012). For the three sites in the United States we basically followed Phillips (1928). However, we also used additional sources for each site as follows: New York City site (Cleaveland, 1866; Adney, 1886; Barrows, 1889); Cincinnati (Wheaton, 1882; Anonymous, 1873; Langdon, 1879; Dawson, 1903); Portland (Pfluger, 1896a; Pfluger, 1896b; Pfluger, 1896c; Pfluger, 1896d; Pfluger, 1896e; Pfluger, 1896f; Pfluger, 1896g; Pfluger, 1897; Jewett & Gabrielson, 1929).

We conducted two sets of tests. In both sets we compared success rates at three levels: all introduced passeriforms; European species only; and the subset of 13 European species introduced to at least one site in New Zealand, Australia and the United States.

In our first set of three tests we compared success ratios across the nine separate sites using contingency tests. In our second set of tests we compared success rates across Australia, New Zealand and the United States using Kruskal–Wallis tests. We emphasize here that this is the scale, albeit crude, at which other studies have been conducted (e.g., Newsome & Noble, 1986; Veltman, Nee & Crawley, 1996; Duncan, 1997; Green, 1997; Sol, 2000).

## Results

For the time span in our study (1850–1900), 50 passeriform species were introduced to at least one of our sites. Of these, 31 were European in origin, and 19 were non-European (Table 1). The species, their fates and origins are presented in Table 1. The total numbers of species introduced to a site ranged from a low of eleven species (New York City) to a high of 27 species in Auckland. In general, the four sites in New Zealand had the most introductions (mean = 24, range 21–27) and sites in the United States had the fewest (mean = 13.33, range 11–16).

**Table 1:** Species introductions arranged by sites within locations.

***Species***	**Vi**	**Sa**	**Ot**	**Cb**	**We**	**Au**	**Ci**	**Pt**	**Ny**
***European species***									
Alauda arvensis*	1	1	1	1	1	1	0	0	0
Carduelis cannabina*	0	0	0	0	0	0	*	0	*
Carduelis carduelis*	1	1	1	1	1	1	0	0	0
Carduelis chloris*	1	1	1	1	*	1	*	0	*
*Carduelis flammea*	*	*	1	1	1	1	*	*	*
*Carduelis flavirostris*	*	*	0	0	*	*	*	*	*
Carduelis spinus*	0	0	*	0	0	*	0	0	*
*Cinclus cinclus*	*	*	*	*	*	*	0	*	*
*Corvus frugilegus*	0	*	*	1	*	0	*	*	*
*Corvus monedula*	*	*	0	0	*	*	*	*	*
*Emberiza cirlus*	*	*	1	*	1	*	*	*	*
Emberiza citrinella*	0	*	1	1	*	1	0	*	*
*Emberiza hortulana*	0	*	*	*	0	*	*	*	*
*Emberiza schoeniclus*	*	*	0	0	*	*	*	*	*
Erithacus rubecula*	0	*	0	0	0	0	0	0	0
Fringilla coelebs*	0	0	1	1	1	1	*	0	0
*Fringilla montifringilla*	*	0	*	0	0	*	*	*	*
*Loxia pytyopsittacus*	*	*	*	*	*	*	*	0	*
*Lullula arborea*	*	*	*	*	*	0	*	0	0
*Luscinia megarhynchos*	*	*	*	*	*	*	0	0	0
*Parus caeruleus*	*	*	*	0	*	*	*	*	*
*Parus major*	*	*	*	*	*	*	0	*	*
*Passer domesticus* ^a^	1	1	1	1	1	1	1	*	1
*Passer montanus*	1	*	0	*	*	0	*	*	*
*Prunella modularis*	*	*	1	1	1	1	0	*	*
Pyrrhula pyrrhula*	*	0	*	0	*	*	0	0	*
Sturnus vulgaris*	1	1	1	1	1	1	0	0	1
*Sylvia atricapilla*	*	*	*	*	*	0	*	0	*
*Sylvia communis*	*	*	*	*	*	0	*	*	*
Turdus merula*	1	1	1	1	*	1	*	0	0
Turdus philomelos*	1	1	1	1	1	1	0	0	0
***North American species***									
*Agelaius phoeniceus*	*	*	*	*	*	0	*	*	*
*Cardinalis cardinalis*	0	*	*	*	*	*	*	*	*
*Mimus polyglottos*	*	*	*	*	*	*	*	0	*
*Piranga rubra*	*	*	*	*	*	0	*	*	*
*Sturnella neglecta*	*	*	*	*	*	0	*	*	*
***Asian species***									
*Acridotheres tristis* ^b^	1	*	0	0	0	*	*	*	*
*Lonchura oryzivora*	0	*	*	*	*	0	*	*	0
*Lonchura punctulata*	*	*	*	*	*	0	*	*	*
*Pycnonotus jocosus*	1	1	*	*	*	*	*	*	*
***Australian species***									
*Gymnorhina tibicen*	*	*	1	1	1	1	*	*	*
*Lonchura castaneothorax*	*	*	*	0	*	0	*	*	*
*Manorina melanocephala*	*	*	0	0	0	*	*	*	*
*Manorina melanophrys*	*	*	*	*	0	*	*	*	*
*Neochmia temporalis*	*	*	0	*	*	0	*	*	*
*Stagonopleura bella*	*	*	*	*	0	0	*	*	*
*Stagonopleura guttata*	*	*	*	0	0	*	*	*	*
*Taeniopygia guttata*	*	*	*	*	0	*	*	*	*
***African species***									
*Euplectes orix*	*	0	*	*	*	*	*	*	*
*Serinus canaria*	0	*	*	*	*	*	*	*	*

**Notes.**

Locations and their sites Australia Vi Victoria Sa South Australia
New Zealand Ot Otago Cb Canterbury We Wellington Au Auckland
United States Ci Cincinnati Pt Portland Ny New York City area

Outcomes are 1 = Successful; 0 = Unsuccessful. Species marked with an asterisk represent the select 13 species released to at least one location inall three regions.

a *Passer domesticus* appeared in Cincinnati in 1869 (Barrows, 1889).

b *Acridotheres tristis* is established now in New Zealand, but from recent releases (Duncan, 1997).

Overall, 17 species succeeded at one or more sites, whereas 33 failed at all sites. This pattern is consistent with the importance of species-level demography demonstrated by a recent modeling study (Cassey, Prowse & Blackburn, 2014). The 50 species were released a total of 170 times. There were 34 releases of 23 species at the two sites in Australia; 96 releases of 41 species in New Zealand; and 40 releases of 22 species at the three sites in the United States (Table 1). The general pattern for species released to more than one site within a region was repeated success or failure. Within regions there were only mixed outcomes for the rook (*Corvus frugilegus*) in New Zealand, and European Starling (*Sturnus vulgaris*) in the United States.

Of the 35 species that were released to more than one site: 18 failed at all sites: five succeeded at all sites: and 12 had mixed outcomes. Nine of the 12 mixed species had mixed outcomes often because they failed at one or more of the sites in the United States (Cincinnati, Portland, or New York). The remaining three species (*Corvus frugilegus*, *Passer montanus*, and *Acridotheres tristis*) were not released at any of the three sites in the United States. We note that *Passer montanus* was introduced successfully to St. Louis, Missouri in 1870 (Barrows, 1889), but was not, as far as we can discern, released at any of the three sites where comprehensive releases occurred in the United States. Also, species such as *Acridotheres tristis* and *Pycnonotus jocosus* are apparently established in Florida (Avery & Moulton, 2007), but neither was released at any of our three sites in the United States, and both were introduced much more recently (*Acridotheres tristiscirca* 1983; *Pycnonotus jocosus* late 1950s, Stevenson & Anderson, 1994).

For the nine species with mixed outcomes, released at one or more sites in the United States, there was a total of 21 failures overall, 18 of these (86%) occurred at one or more of the three sites in the United States. Within regions, only *Corvus frugilegus* (New Zealand) and *Sturnus vulgaris* (United States) had mixed outcomes (Table 1). Of course, *Sturnus vulgaris* is now widespread and abundant throughout the United States and southern Canada. However, in the Portland (Oregon) area, a release of 35 pairs of *Sturnus vulgaris* “in 1889 and 1892” (Pfluger, 1896e), apparently was unsuccessful. Lord (1902, page 239) reported seeing some Starlings (*Sturnus vulgaris*) in Portland, Oregon in 1901 and Jewett & Gabrielson (1929) quoted Pfluger (1896e) in saying the birds “...have since increased remarkably well”. However, the release by Pfluger (1896e) evidently failed as Jewett (1946) later reported on the “first record of occurrence” of *Sturnus vulgaris* in Oregon as occurring in 1943, contradicting the speculation that the earlier release succeeded.

**Figure 1:**
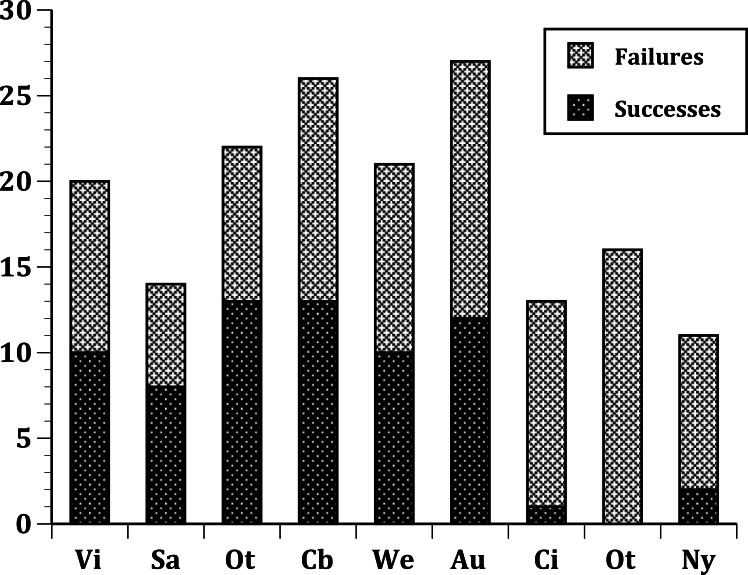
Plot of the number of successful and unsuccessful species introductions for all passeriform species introduced to 9 sites. (United States) Nyc, New York City; Cin, Cincinnati, Ohio; Ptl, Portland, Oregon; (Australia) Soa, South Australia; Vic, Victoria, Australia; (New Zealand) Wel, Wellington; Ota, Otago; Auc, Auckland; Cby, Canterbury.

**Figure 2:**
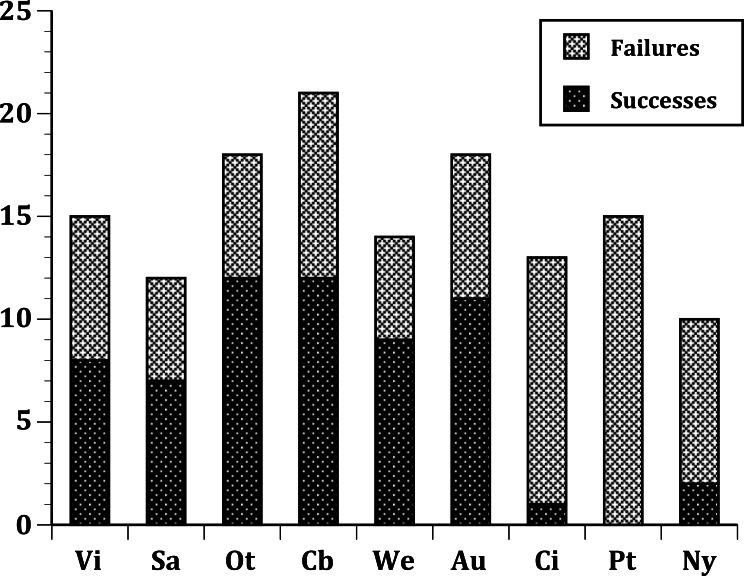
Same as Fig. 1, but limited to just species with a European origin introduced to 9 sites.

**Figure 3:**
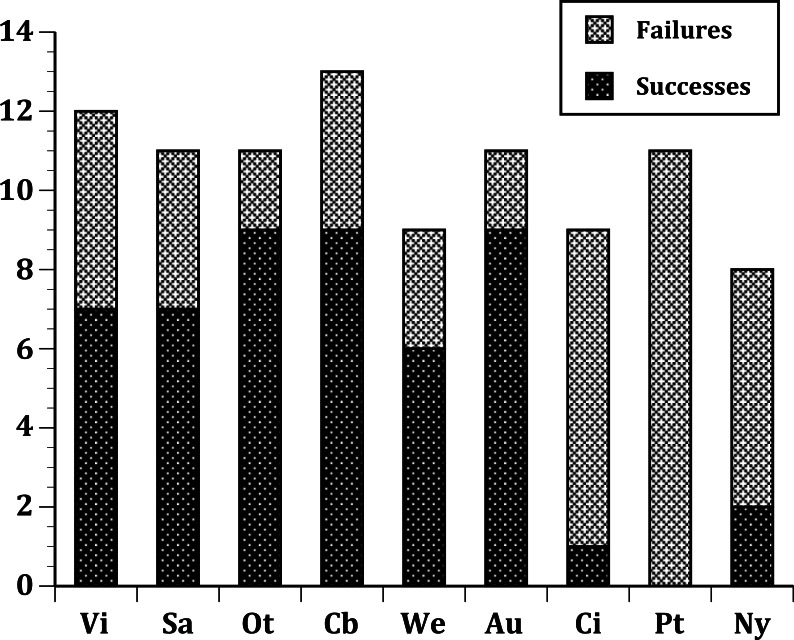
Same as Fig. 2, but further limited to include just the 13 select European species introduced into all three regions: Australia; New Zealand; and the United States.

In our first set of tests, we found that the nine sites differed significantly at all three levels: (1) all species (Fig. 1); (2) European species only (Fig. 2); (3) select 13 species released (Fig. 3) at all sites (Table 2). In the second set of comparisons, comparing success rates across regions, the difference when all species were included was showed a clear, but not significant trend (Kruskal–Wallis *χ*^2^ = 5.67, *p* > *χ*^2^ = 0.06). However, when we restricted the comparison to just those species from Europe, success rates differed significantly (Kruskal–Wallis *χ*^2^ = 6.3, *p* > *χ*^2^ = 0.04), and in our final test, which included just the 13 select European species introduced to all three regions, success rates were also significantly different (Kruskal–Wallis *χ*^2^ = 7.1, *p* > *χ*^2^ = 0.03).

**Table 2:** Results of contingency tests comparing passeriform introduction success rates across nine sites.

Group	*χ* ^2^	*p* < *χ*^2^
All species	27.27	0.0006
European only	33.42	0.00005
Select European	31.25	0.0001

Of the 26 European species released to more than one site, 12 failed at all sites, three succeeded at all sites, and 11 had mixed outcomes (Table 1). In general, European species exhibited high predictability for success, with three species always succeeding (*Passer domesticus*,*Carduelis flammea*, *Emberiza cirlus*) and others succeeding 6 out of 9 times (*Alauda arvensis*, *Turdus philomelos* and *Carduelis carduelis*), five out of six times (*Carduelis chloris*), five out of seven times (*Turdus merula*), seven out of nine times (*Sturnus vulgaris*), and four out of five times (*Prunella modularis*). Four species demonstrated somewhat lower predictability: *Passer montanus* and *Corvus frugilegus* each succeeded at one of three sites; *Fringilla coelebs* succeeded at four of eight sites; and *Emberiza citrinella* succeeded at three of five sites. Of these latter four species, only *Passer montanus* was successful in a region other than New Zealand. Species with mixed outcomes mostly failed in the United States, and the species that failed tended to have high success rates in Australia and/or New Zealand. Moreover, not a single species succeeded just at one of the sites in the United States: all were also successful either in New Zealand or Australia or both.

## Discussion

Our results indicate that success rates of introduced passeriform birds differed significantly across the three regions and among sites within those regions. Moreover, the main reason for these differences involves the low rate of success for introductions at the three sites in United States. Such differences may not be easily detected because overall success rates for introductions tend to be very low (e.g., Long, 1981; Blackburn, Cassey & Lockwood, 2009).

Differences in the overall introduction success rates in different locations may simply be a result of differences in colonization pressure (Lockwood, Cassey & Blackburn, 2009). Indeed, the historical record for the three regions here shows clearly that where there were more species introduced (i.e., New Zealand) there were more successfully established species.

Location-level comparisons could lose validity when they include heterogeneous sets of species (Sol, 2000). Limiting our analyses to passeriform birds partially corrects for any such influence, but of course, heterogeneity might be expressed also in the origin of the species. Thus, species from one part of the world might tend to have higher or lower success rates than species form other areas. In our tests this is seen in the three different levels of our regional-level comparisons. For example, for the 19 species that did not originate in Europe, there were 34 total releases of which nearly 80% (27) were unsuccessful. In some cases this is likely due to the small number of sites to which species from regions other than Europe were released (Moulton & Sanderson, 1997). Nevertheless, 31 of the 50 species (62%) in our study were European in origin. These European species were released a total of 136 times of which 62 were successful and 74 unsuccessful, whereas for non-European species there were seven successful and 27 unsuccessful introductions (Table 1). These ratios are significantly different (RXC test, *χ*^2^ = 7.05, *p* > *χ*^2^ < 0.008). This is mostly the result of poor introduction success rates of the eight Australian and two African species in this study. Apart from our historical and regional limitations, the four Asian species (*Acridotheres tristis, Lonchura punctulata; Lonchura oryzivora*; and *Pycnonotus jocosus*) all are characterized by having very high introduction success rates over all their releases (Sol et al., 2012).

If species with relatively lower success rates were released more at sites in the United States than at sites in Australia or New Zealand, any comparison that included sites where these species were not released might lead to a biased outcome. Such is not the case here as the sites in the United States, had only two species’ introductions that were not from Europe: *Mimus polyglottos* (a North American species) to Portland, and *Lonchura oryzivora* (an Asian species) to New York City.

A second concern might be raised because we limited our analyses to introductions in the 19th century, and to selected sites based on their being major centers for passeriform introductions. However, our timing restriction is reasonable since this is when the acclimatization societies were most active: New Zealand and Australia starting around 1860 (Moulton, Cropper & Avery, 2011; Moulton et al., 2012), New York City in the 1850s, Cincinnati in the early 1870s (Anonymous, 1873), Portland in the late 1880s (Jewett & Gabrielson, 1929).

Like other patterns gleaned from historical records (e.g., studies of propagule pressure) the underlying cause or causes are not clear. As we have shown elsewhere (Moulton, Cropper & Avery, 2011; Moulton et al., 2012; Moulton, Cropper & Avery, 2012; Moulton, Cropper & Avery, 2013; Moulton & Cropper, 2014), propagule pressure is not likely the best explanation of the pattern of introduction success in these regions, despite the arguments posed by Blackburn et al. (2011) and Blackburn et al. (2013). Indeed, in a population model analysis Cassey, Prowse & Blackburn (2014) found that net reproductive rate (*R*_0_) was a more important determinant of introduction outcome than propagule pressure for three hypothetical species. The realized net reproductive rate in novel environments is likely to be greatly influenced by habitat suitability. Moreover for one of the sites in the United States (Cincinnati) we have no information on the numbers of individuals released per species.

We have made an effort here to choose species with shared taxonomic and biogeographic affiliations, reducing but not eliminating, possible species-level factors such as those described by Blackburn, Lockwood & Cassey (2009), Blackburn, Cassey & Lockwood (2009), Sol et al. (2005) and Sol et al. (2012).

Following the invasion pathway model of Duncan, Blackburn & Sol (2003) our results suggest that location-level factors are an important factor in determining which introductions succeed. It is unknown why introductions to the United States in this time frame experienced lower success rates. It is possible that the physical environment at the three sites in the United States is simply harsher when compared to sites in Australia or New Zealand. It is also possible that interspecific competition with native species, predation or some combination of these forces and characteristics of the natural environment could be operating.

There are at least two possible alternatives to location-level factors in generating the pattern of reduced introduction success in the United States. These involve differences at the individual-level and differences related to the skill of the people introducing the birds.

At the individual-level, it is not difficult to imagine that the individual birds in the releases to the United States might have been severely stressed having arrived by ship after a lengthy voyage. The actual length of the voyage is only one part of the process here. Individuals involved in the releases might have been held in captivity for lengthy periods of time. Thus, the amount of time needed for the whole process of obtaining, transporting and releasing the birds could affect the condition of the individuals in the shipments (Blackburn, Lockwood & Cassey, 2009). As noted above, the death rate among birds during transport in the 19th century was staggering (see Thomson, 1922; Balmford, 1978; Phillips, 1928 for examples). Transport and handling conditions could well have significantly impacted the health and physical condition of the surviving individuals.

However, in contrast to the lengthy voyages too New Zealand as noted above, ocean transport to New York from England as early as the 1850s may have taken as little as 12 days (Gibbs, 1952). Thus, if transport times alone were the cause of reduced success rates, we would predict that New Zealand sites would have the lowest success rates–not the highest. We do not, however, have exact information on transport time to Portland, Oregon from New York, but in 1870 it took seven days by rail to go from New York to San Francisco (about 4700 km– http://www.shmoop.com/transcontinental-railroad/statistics.html), and it would be difficult to imagine the rail journey from San Francisco to Portland taking more than a few days (about 1024 km). The distance from New York to Cincinnati (1025 km) is almost the same as San Francisco to Portland, Oregon.

We note that the birds sent to Cincinnati and Portland were identified as “German birds” although it is unknown whether the individuals actually came from Germany or were simply birds native to Germany that originated somewhere else. For shipments from Germany to Portland and Cincinnati we do not know what travel interruption times were, nor can we document the level of care the birds received while caged before release.

The second possibility involves differences in the level of skill among the people conducting the introductions. Lamb (1964) detailed variability in transport conditions to New Zealand in the nineteenth century depending on who tended the birds in transit, as well as on the features of the ships themselves. Unfortunately, we have no way of evaluating the skill levels of people introducing birds at the site in the United States. It is unlikely, but not impossible, that the level of incompetence needed to produce the patterns seen here would be so non-randomly distributed.

Unfortunately, the historical record does not allow us to differentiate clearly between the effects of location-level factors and the alternatives of individual-level factors and skill, or lack thereof, of the people who introduced the birds. However, we note that Simberloff & Gibbons (2004) listed examples of presumably established introduced species whose populations crashed and that sometimes vanished. These included *Acridotheres cristatellus* in the vicinity of the city of Vancouver (Colautti & MacIsaac, 2004) and *Leiothrix lutea* in the Hawaiian Islands (Ralph et al., 1998). To this list we can add *Alauda arvensis* on the western end of Long Island (Eaton, 1914; Phillips, 1928). Despite the conclusion that the dramatic decreases of these species were “mysterious” (Simberloff & Gibbons, 2004) it is not difficult to imagine, at least where development is intensive, that environmental or habitat changes influenced the declines. Increasingly, it is becoming clear that introduction success is not a simple function of the number of individuals introduced, but perhaps can best be explained as an interaction of site-specific and species-specific factors (Bacon et al., 2014) coupled with additional event-level influences.
